# MicroRNAs in the Host-Apicomplexan Parasites Interactions: A Review of Immunopathological Aspects

**DOI:** 10.3389/fcimb.2016.00005

**Published:** 2016-02-02

**Authors:** Carla C. Judice, Catarina Bourgard, Ana C. A. V. Kayano, Letusa Albrecht, Fabio T. M. Costa

**Affiliations:** ^1^Laboratory of Tropical Diseases, Department of Genetics, Evolution and Bioagents, University of CampinasUNICAMP, Campinas, Brazil; ^2^Instituto Carlos Chagas, Fiocruz-ParanáCuritiba, Brazil

**Keywords:** microRNA, immune response, cell host, Apicomplexa, parasites

## Abstract

MicroRNAs (miRNAs), a class of small non-coding regulatory RNAs, have been detected in a variety of organisms ranging from ancient unicellular eukaryotes to mammals. They have been associated with numerous molecular mechanisms involving developmental, physiological and pathological changes of cells and tissues. Despite the fact that miRNA-silencing mechanisms appear to be absent in some Apicomplexan species, an increasing number of studies have reported a role for miRNAs in host-parasite interactions. Host miRNA expression can change following parasite infection and the consequences can lead, for instance, to parasite clearance. In this context, the immune system signaling appears to have a crucial role.

## Introduction

MicroRNAs (miRNAs) are small non-coding RNAs from 18 to 24 nucleotides in length, initially described in *Caenorhabditis elegans* (Lee and Ambros, 2001). They are involved in gene expression regulation by binding to mRNAs and affecting the translation process (Ambros, 2004). In a canonical way, miRNAs derive from a long primary transcript, which is processed into a short precursor (pre-miRNA) by the Drosha enzyme complex. The pre-miRNA has around 70 nucleotides and is exported into the cytoplasm by exportin-5. In the cytosol, RNAse III Dicer recognizes and cleaves the hairpin loop of the pre-miRNA. One strand of the mature miRNA duplexes associates with the RNA Induced Silencing Complex to form a miRNA- ribonucleoprotein complex. Then, it binds to the target sites of mRNAs, predominantly in the 3′- end untranslated region (UTR) of the target mRNA for translational repression or mRNA cleavage (Lewis et al., 2005; Bartel, 2009) (Supplementary Figure). Since miRNAs often target many different mRNAs, an individual miRNA can have a wide range of regulatory functions (Kim, 2005). miRNAs have been found in all Metazoa studied so far, and are implicated in many cellular processes such as developmental timing, cell proliferation and death (Ambros, 2004; Bartel, 2004). In humans, more than 700 miRNAs have been identified and it is hypothesized that they affect translation of almost 30% of human genes (Bartel, 2004; Kim and Kim, 2007).

The importance of miRNAs has been described in many parasitic pathologies. In schistosomosiasis, a worldwide disease caused by the trematode worm *Schistosoma*, it have been shown that miRNAs can regulate development of the parasite and also the hepatic pathology and several signaling pathways in *Schistosoma*-infected mice (Zhu et al., 2014). *Leishmania*, the causing agent of leishmaniasis, has been shown to target Dicer1 and to downregulate miR-122 which in consequence result in an induced liver parasite burden (Ghosh et al., 2013). Furthermore, alterations in cell host miRNAs have also been described in macrophages and dentritic cells infected with *Leishmania* (Frank et al., 2015; Geraci et al., 2015). In Chagas disease, caused by the parasite *Trypanosoma cruzi*, the involvment of miRNAs on resulting cardiovascular disorders have been recently reported (Ferreira et al., 2014; Linhares-Lacerda et al., 2015; Navarro et al., 2015).

The presence of these small regulatory non-coding RNAs has also been described for the Apicomplexa parasites. This phylum comprises phylogenetically related taxa (around 5000 species), the majority of which are obligate intracellular parasites affecting many animal species and responsible for diseases that significantly impact upon human health (Cavalier-Smith, 1993). The Apicomplexa include *Plasmodium falciparum* and *Plasmodium vivax*, amongst others, the major causal agents of human malaria (World Health Organization, 2015); *Toxoplasma gondii*, the causative agent of toxoplasmosis (Lüder and Gross, 2005) and *Cryptosporidium parvum*, a waterborne agent of cryptosporidiosis, responsible for outbreaks worldwide (Guerrant, 1997). Once inside the host, parasites may regulate gene expression in host cells to improve their abilities to infect and proliferate inside their respective target cells, such as epithelial cells (Deng et al., 2004; Amino et al., 2008; Mcdonald et al., 2013), hepatocytes (Prudêncio et al., 2006; Sturm et al., 2006), erythrocytes (Michel et al., 1980; Gazzinelli et al., 2014) and, for some species, specialized immune cells such as macrophages and dendritic cells (Leng et al., 2009). Usually, this occurs via inhibition of host immune responses, including those involved in apoptosis and cytokine production (Plattner and Soldati-Favre, 2008). A growing body of evidence has demonstrated that parasites promote modifications on host miRNA population, underscoring the importance of miRNAs in parasite-host interactions. Here we review the latest evidence for alterations induced on host miRNAs by Apicomplexa parasites (e.g., *Cryptosporidium, Plasmodium* and *Toxoplasma*) focusing on those related to the host's immune response.

### Cryptosporidium

A growing number of functional studies have reported the role of miRNAs in the human host response to *Cryptosporidium*. Evidence from *in vitro* and *in vivo* studies indicates that both innate and adaptive immunity are implicated in the resolution of cryptosporidiosis and resistance to infection (Akira et al., 2004; Chen et al., 2005). Several miRNAs involved in the Toll-like Receptor 4 (TLR-4) and NF-κB signaling pathways have been well documented in *C. parvum* infection. Using an *in vitro* model of human cryptosporidiosis, Zhou et al. analyzed *C. parvum*-induced miRNA expression in cholangiocytes. The infection was able to induce expression of a series of miRNAs and among them mir-125b-1, mir-21, mir-30b, and mir-23b-27b-24-1 cluster genes were transactivated through promoter binding of the NF-κB p65. Functional inhibition of these miRNAs subset resulted in an increased *C. parvum* burden (Zhou et al., 2009). Human cholangiocytes infected with *Cryptosporidium* also showed downregulation of let-7 in a NF-κB-dependent mechanism. As a consequence, there was upregulation of TLR 4 (let-7 target) and a better epithelial defense response against the parasite (Chen et al., 2007). Moreover, let-7 act together with miR-98 to control expression of suppressors of inflammatory cytokine signaling (SOCS/CIS) and proteins (Hu et al., 2010). Induction of CIS expression enhances IκBα degradation resulting in NF-κB transcription factor activation. Conversely, negative feedback regulation of TLR4/NF-κB signaling may be reached by miR-21 induction after *C. parvum* infection, as miR-21 targets PDCD4, a proinflammatory protein that promotes activation of NF-κB and suppresses interleukin 10 (Sheedy et al., 2010).

In the context of regulation of TLR4/NF-κB-mediated epithelial responses, miR-27b directly targets KSRP4 and modulates NOS2 (inducible) mRNA stability following *C. parvum* infection (Zhou et al., 2012). Moreover, histone deacetylases (HDACs) and NF-κB signaling have been described as regulators of mir-424 and miR-503 suppression, which in turn promote mucosal antimicrobial defense (Zhou et al., 2013).

In terms of cellular adhesion, *C. parvum* infection resulted in decreased miR-221 expression in infected epithelial cells. Moreover, intercellular adhesion molecule-1 (ICAM-1) was described as miR-221 direct target. Downregulation of miR-221 is probably involved in increased infiltration of lymphocytes into the intestinal mucosa (Gong et al., 2011).

### Toxoplasma

Initial host global miRNA expression approaches for *Toxoplasma gondii* showed that the parasite specifically modulates expression of important host miRNAs during infection (Zeiner et al., 2010). After 24 h, *T. gondii* infection altered around 14% of host miRNAs in primary human foreskin fibroblasts, which could be related to the NF-κB activation signaling (Shapira et al., 2002). Upregulation was confirmed for the host primary transcripts miR-17~92 and miR-106b~25 that are known to play crucial roles in mammalian cell cycle regulation. In particular, in adult animals miR-17~92 and miR-106b~25 have been shown to influence the functionally intertwined pathways of apoptosis and G1/S cell cycle progression by targeting multiple components of each pathway (Xiao and Rajewsky, 2009).

NF-κB signaling and transactivation by STAT3 binding was demonstrated to regulate a subset of miRNAs (miR-30c-1, miR-125b-2, miR-23b-27b-24-1, and miR-17 ~ 92 cluster genes) that were induced under *T. gondii* infection in human macrophages. These miRNAs are mainly involved in anti-apoptosis in response to *T. gondii* infection (Cai et al., 2013). Recent study highlighted two immunomodulatory miRNAs, miR-146a and miR-155, important for the cell host response to *T. gondii* challenge. Both were induced in mice brains under *T. gondii* infection, but in a strain-specific manner (Cannella et al., 2014). Mice challenged with *T. gondii* cystogenic (type II) strain showed an exclusive and significant induction of miR-146a, a key immune and inflammatory response regulator targeting IRAK1 and TRAF6 (Taganov et al., 2006; Saba et al., 2014). The data indicated that type II allele ROP 16 (rhoptry protein 16) lacked the miR-146a suppression activity observed for type I allele. ROP16 is a phosphotyrosine kinase released from the *Toxoplasma* rhoptries that activates STAT3/6 signaling pathways in the host cell nucleus (Saeij et al., 2007). Another immunoregulatory miRNA, miR-155 (Faraoni et al., 2009; Vigorito et al., 2013), was induced by all tested strains. Comparative analysis among strains demonstrated that ablation of miR-146a affects early parasite burden, leading to significant differences in interferon (IFN)-γ production and in long-term survival of mice.

Studies of miRNAs as biomarkers in *T. gondii* infection have provided potential candidates. A recent study investigated a correlation between plasma miRNA levels and *T. gondii* infection (Jia et al., 2014) comparing miRNA expression profiles from *T. gondii*-infected mice with healthy mice. Among the up-regulated miRNAs, three of them (mmu-miR-712-3p, mmu-miR-511-5p, and mmu-miR-217-5p) kept the induced expression in infected mice with either RH or ME49 strain of *T. gondii*. Moreover, the up-regulation of these miRNAs was shown to be a specific response to *T. gondii* infection, as challenge with other pathogens such as *Plasmodium berghei, P. yoelii, P. chabaudi* and *C. parvum*, resulted in down-regulation of these miRNAs. The parasite-specificity of miR-712-3p, miR-511-5p, and miR-217-5p make them good biomarkers for *T. gondii* infection.

### Plasmodium

#### In the vertebrate host

Recent studies focusing on miRNA pathways in *Plasmodium falciparum* have shown a lack of ortholog candidates to the main components of Dicer complex and RISC in the *P. falciparum* genome (Hall et al., 2005). Sequencing and bioinformatics analysis of small RNA libraries from *P. falciparum* infected erythrocytes (Pf-iE) were not able to identify parasite-specific miRNAs (Rathjen et al., 2006). Instead, the presence of high levels of human miR-451 in both infected and healthy red blood cells (RBC) was reported, leading the authors to suggest that miR-451 could be functional in the differentiation of erythroid cells. Further studies also reported the lack of *Plasmodium*-specific miRNAs. Curiously, miR-451 was once again detected in RBCs, with higher expression in Pf-iE (Xue et al., 2008). These data support the genome lack of Dicer and Argonaute orthologues in *P. falciparum*, crucial enzymes in miRNAs biogenesis (Coulson et al., 2004; Hall et al., 2005).

A better understanding of the miR-451 role in Pf-iE came from a study using sickle cell (HbS) erythrocytes. In the context of malaria, a well-established resistance to infection is associated with this specific cell type (Cholera et al., 2008; Cyrklaff et al., 2011; Ferreira et al., 2011). Recently, La Monte and colleagues found a role for miRNAs from HbS erythrocytes in resistance against malaria (Lamonte et al., 2012). For the first time, translocation of human miRNAs into the parasite was characterized, with around 100 human miRNAs detected within parasites. In particular, miR-451 and let-7i were shown to be enriched in HbAS and HbSS erythrocytes. The miRNA uptake profile across the intraerythrocytic developmental cycle was examined to show that let-7i exhibited high expression after 16 h of parasite growth, whereas expression of miR-451 peaked after 32 h, indicating a dynamic uptake of miRNAs. Moreover, the authors demonstrated that these two miRNAs were able to form chimeric fusions with *P. falciparum* mRNAs which implies translational inhibition via impaired ribosomal loading. Integration of miR-451 into *P. falciparum* regulatory PKA (cAMP dependent kinase) transcripts was shown. PKA-R is crucial to parasite survival (Wurtz et al., 2011) and its suppression mediated by miR-451 was related to an increased number of gametocytes. In addition, administration of miR-451 and miR-223 analog molecules resulted in a significant reduction in the growth of *P. falciparum* (46%). This study provided the first data on human miRNAs regulating *Plasmodium* gene expression and suggested the possibility of miRNAs being incorporated into malaria parasites. Moreover, a very recent study investigated plasma miRNAs alterations mediated by *P. vivax* and showed downregulation of miR-451 and miR-16 in *P. vivax* malaria patients (Chamnanchanunt et al., 2015).

Changes in miRNAs expression profile have also been evaluated in experimental malaria models. Modifications in liver miRNAs were initially investigated in mice infected with self-healing *P. chabaudi* malaria (Delić et al., 2011). Here primary infections, but not secondary infections, could induce upregulation of hepatic mRNAs related to the immune response (such as IL-1β, TNF-α, IFN-γ, and NF-κB) and promote alterations in liver miRNAs. Changes in hepatic miRNAs usually associated with adaptive immune responses were detected: miR-26b, MCMV-miR-M23-1-5p, and miR-1274a were found upregulated and 16 miRNA species (miR-101b, let-7a, let-7g, miR-193a-3p, miR-192, miR-142-5p, miR-465d, miR-677, miR-98, miR-694, miR-374^*^, miR-450b-5p, miR-464, miR-377, miR-20a^*^, and miR-466d-3p) were downregulated. The expression level of the miRNAs related to the immune response remained unchanged for almost all of them in re-infected mice. Although the data did not explain the mechanisms underlying the changes in miRNAs expression, they appear specific for malaria infection and important in acquired protective immunity against *P. chabaudi*. A recent study reported very significant upregulation of miR-155 in liver after infection with genetically attenuated parasites (GAP) (Hentzschel et al., 2014). Another immuno-regulatory miRNA, miR-21 was also shown to be induced in infected GAP mice. Additionally, GAP injection also induced TNF-α and IFN-γ expression, two known upstream regulators of miR-155. The crucial relevance of miR-155 in *Plasmodium*-infected liver was demonstrated when the ectopic administration of miR-155 (AAV-155) reduced the number of GAP injections necessary to achieve immunity in mice.

The pathogenesis of experimental cerebral malaria (ECM) is multifaceted and evidence suggests that the host immune system plays a major role in expression of certain cytokines. Immune modulation (Hunt and Grau, 2003), apoptosis (Lackner et al., 2007), leukocyte cytoadhesion (Baptista et al., 2010; Costa et al., 2011), and possibly hypoxia (Penet et al., 2005) are also involved. El-Assad *et al* compared expression levels of selected miRNAs related with the processes above: let-7i, miR-27a, miR-150, miR-126, miR-210, and miR-155 (El-Assaad et al., 2011). Mice with cerebral malaria infected with *P. berghei* ANKA were compared to mice infected with non-cerebral malaria strains (Pb-K173 or PbK) and let-7i, miR-27a, and miR-150 were shown to be upregulated in brain tissue of *P. berghei* ANKA infected mice. While let-7i belongs to the let-7 miRNA family previously described to control cellular proliferation and the innate immune response (O'Hara et al., 2010), miR-150 is highly expressed in monocytes and is related to cell proliferation and apoptosis. The authors suggested that miR-150 could be controlling monocyte accumulation in microvasculature, one of the features of fatal ECM. miR-27a is involved in apoptosis induction, increased TNF sensitivity, regulation of T cell proliferation and the NF-κB signaling pathway during inflammation (Chhabra et al., 2009; Tourneur and Chiocchia, 2010). Its upregulation was only observed with the PbA strain (PbK was similar to the naïve control), suggesting a specific role for this miRNA in the neurological ECM syndrome.

Taken together, these studies demonstrate the importance of miRNAs in the host response to *Plasmodium* infection and strongly suggest that a reprogramming of miRNA expression could have a regulatory function in malaria pathogenesis.

#### In the invertebrate host

The malaria parasite-vector interaction has received much attention as this is a great target step to interrupt and/or lessen the burden of pathogen transmission (Biron and Loxdale, 2013). An important bottleneck suffered by *Plasmodium* spp. during its life cycle occurs when parasites go through the gut of *Anopheles* spp. mosquito vectors (Cirimotich et al., 2010). It was demonstrated that defense responses from *Anopheles* spp. beside includes various aspects of innate immunity (systemic humoral immunity, cell adhesion, redox metabolism and detoxification), and also extracellular-matrix remodeling, intracellular local epithelial reactions from the midgut epithelial cells, and apoptosis (Vlachou et al., 2005). So, comprising a number of mosquito factors that have been shown to affect development of *Plasmodium* parasites in the invertebrate host (Blandin et al., 2008; Cirimotich et al., 2010).

Initially, several putative miRNAs of *Anopheles gambiae*, the most important African malaria vector, were reported based on similarity to known miRNAs that are conserved in *Drosophila* spp. (Lai et al., 2003; Wang et al., 2005; Chatterjee and Chaudhuri, 2006). The first study of isolated miRNAs from *P. berghei* infected midguts was able to identify 18 miRNAs in *A. gambiae* (Winter et al., 2007). Aga-miR-34, aga-miR-1175, and aga-miR-1174 were downregulated in infected blood-fed midgut samples. On the other hand, aga-miR-989 was induced by infection. Additionally, Drosha, Dicer I and Argonaute 1 mRNAs silencing promoted better parasite survival. Overall, these data support an involvement of miRNAs as key players in the regulation of *Anophele*s resistance against *Plasmodium* invasion and survival (Winter et al., 2007).

By using next generation sequencing, Jain *et al*. identified and validated 126 miRNAs in post-blood feeding and infection in *A. stephensi*, of which 16 and 13 were regulated during feeding with parasite-infected or non-infected blood, respectively (Jain et al., 2014). Upon parasitized blood feeding, a tight-controlled miRNA expression was observed, suggesting a role during the gonotrophic cycle in the mosquito. Analysis of miRNA expression revealed several metabolic pathways as targets for miRNA regulation, including redox homeostasis and protein processing machinery components. Most importantly, some miRNAs (miR-124, miR-305, and miR-309) were identified to target several genes of immune pathways (Jain et al., 2014). In a comprehensive analysis of miRNAs in *A. gambiae*, Biryukova et al. described species-specific production of dominant mature miRNAs induced by blood feeding (miR-7, miR-92a, miR-317, and miR-N3) and by parasitized blood feeding (miR-317 and miR-2940) (Biryukova et al., 2014).

Finally, although several miRNAs have been identified, scarce information is available regarding their expression profile in different stages of parasite maturation within the host. Further investigation on miRNA roles in vector-parasite interaction may contribute to a better understanding of parasite virulence attenuation, which in turn could help hamper malaria transmission.

## Conclusions

**Figure 1:**
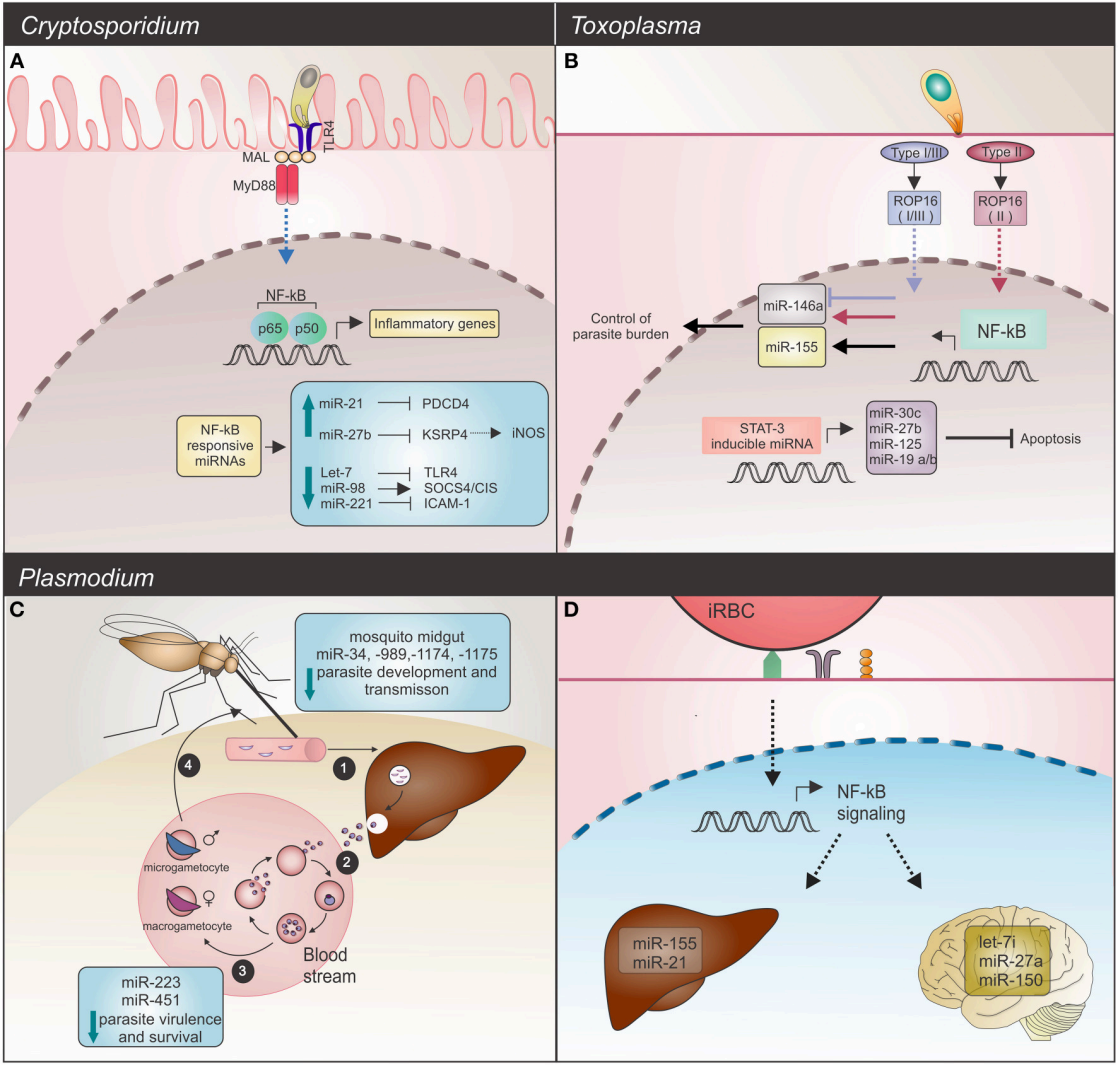
**Host miRNAs response upon Apicomplexa parasite challenge. (A)** *C. parvum* infection: in infected mice epithelial cells, the parasite alters NF-κB-responsive miRNAs. Induction of expression, observed for miR-21 and miR-27b blocks their respective mRNAs, PDCD4 and KSPR4. Downregulation of KSRP4 modulates iNOS levels (dashed arrow). Decreased levels of let-7 and miR-221 promote upregulation of the TLR4 and ICAM-1 mRNAs, while downregulation of miR-98 results in SOCS4/CIS increased levels. These host miRNAs promote regulation of the TLR/NF-κB signaling and also target NF-κB-regulated immune or inflammatory genes. TLR4 (toll-like receptor 4); MAL (MyD88-adapter-like); MyD88 (myeloid differentiation primary response gene 88); PCDP4 (programmed cell death protein 4); KSRP4 (KH-type splicing regulatory protein 4); ICAM-1 (intercellular adhesion molecule 1); NOS2 (inducible nitric oxide synthase 2). **(B)** *T. gondii* infection: common and strain-specific mice miRNA expression is mediated by NF-κB signaling and STAT3 transactivation. The STAT3 transcription factor binds and regulates expression of miR-30c, miR-27b, miR-125, and miR-19, which in turn results in an anti-apoptosis response. Strain-specific miRNA expression could also be detected. Type I, II, and III strains are able to induce miR-155 (black arrow) whereas only the type II strain induces miR-146a expression (red arrow). Strains expressing type I or III ROP16 alleles suppress miR-146a (blue arrow). Deficiency of this miRNA is related with a better control of parasite burden and long-term survival in infected mice. **(C,D)** *Plasmodium* infection. **(C)** Development cycle of *Plasmodium* spp. begins with (1) sporozoites, the parasite forms injected into the vertebrate host skin by a mosquito, invasion of liver and development of (2) merozoites, forms that infect erythrocytes. (3) Male and female gametocytes are then generated by some intra-erythrocytic parasites and (4) taken up by a mosquito. Alterations in mosquito miRNAs occur as a defense mechanism against parasite invasion, resulting in reduced parasite development and transmission. On the other hand, in the human host bloodstream, *P. falciparum* infected sickle cells show upregulation of crucial miRNAs for parasite growth control. High levels of miR-451, let-7, and miR-223 negatively affect parasite growth. **(D)** In mice, potential regulatory roles for miRNAs in experimental severe malaria are highlighted by let-7i, miR-27a, and miR-150 (in brain), which were induced by *P. berghei* challenge. Endothelial liver cells upon *P. chabaudi* infection also display miRNA alterations, as evidenced by upregulation of miR-21 and miR-155.

The growing number of articles reporting host miRNAs changes after parasite infection challenge demonstrates the importance of these non-coding small RNA molecules for the host response. A comparative analysis between the Apicomplexan parasites *Toxoplasma, Cryptosporidium* and *Plasmodium* revealed a huge diversity of miRNAs involved in the host response (Figure 1).

The only miRNA to date whose association with *Cryptosporidium, Toxoplasma* and *Plasmodium* infections has been validated by functional assays is let-7 (Chen et al., 2007; Iliopoulos et al., 2009; Zeiner et al., 2010; Delić et al., 2011). Specific characteristics such as host cell type and the particular biology of the parasite contribute to expression of different host miRNAs. Some convergence on host responses can be found on miRNAs associated with mammalian immunity (Table 1). All the listed miRNAs are related to NF-κB signaling. Some are common miRNAs among these parasites and others are apparently specific, such miR-223 (Gazzinelli et al., 2014). For instance, miR-146 was first identified as an immune system regulator and acts on the mammalian response to microbial infection (Baltimore et al., 2008). Expression of mature miR-146 species is responsive to LPS and proinflammatory cytokines, like TNF (Taganov et al., 2006; Hou et al., 2009). Its expression was shown to be induced after *Plasmodium, Cryptosporidium* and *Toxoplasma* infections. Even though miR-155 had been described as non-responsive to *Cryptosporidium* infection, it is induced upon *Toxoplasma* and *Plasmodium* challenges (Zhou et al., 2009; Lamonte et al., 2012; Cannella et al., 2014; Hentzschel et al., 2014). This miRNA is considered an immune-response molecule sensitive to cytokines and regulated by NF-κB and the Jnk kinase (O'Connell et al., 2010; Elton et al., 2013). It has been considered part of the innate immune response because of its increased expression in macrophages under inflammatory conditions (Tili et al., 2007; Ceppi et al., 2009; Kohlhaas et al., 2009; Tang et al., 2010). However, some studies have suggested that miR-155 is also involved in the adaptive immune response. Upregulation of miR-155 has been described in activated B and T (CD4^+^) cells, although it may be also connected to cytokines (Yin et al., 2007; Banerjee et al., 2010; Lind and Ohashi, 2014).

**Table 1:** **miRNAs responsive to Apicomplexa parasites infection**.

**miRNA**	**Target mRNAs or signaling pathways**	**Apicomplexa parasites**			**References**
		***Cryptosporidium***	***Toxoplasma***	***Plasmodium***	
let-7	TLR-4; IL-6	√	√	√	Chen et al., 2007; Jain et al., 2014
miR-146	IRAK1-2; TRAF6	√	√	√^*^	Iliopoulos et al., 2009; Cannella et al., 2014
miR-155	MyD88; TAB2; IKKε; FOXP3; SOCS1		√	√^*^	Taganov et al., 2006; Ceppi et al., 2009; O'Connell et al., 2010; Tang et al., 2010; Elton et al., 2013; Hentzschel et al., 2014
miR-106b	IL-10	√	√	√^*^	Kohlhaas et al., 2009
miR-27	KSRP; NOS2; PPARγ	√	√	√^*^	Kuehbacher et al., 2007; Baptista et al., 2010
miR-223	TLR3; TLR4; IKKKα			√	Chen et al., 2007; Rénia et al., 2012
miR-98	SOCS	√			Hu et al., 2009

* *Indicates data from microarray studies without a functional assay. TLR, Toll-like receptor; IL, interleukin; IRAK, IL-1R-associated kinase; TRAF6, TNFR-associated factor 6; MYD88, myeloid differentiation primary-response protein 88; TAB2, TAK1-binding protein 2; IKK, inhibitor of NF-κB kinase; FOXP3, forkhead box P3; SOCS1, suppressor of cytokine signaling 1; KSRP, KSRP, KH-type splicing regulatory protein; NOS2, inducible nitric oxide synthase 2; PPARγ, peroxisome proliferator-activated receptor-γ*.

Expression of miR-106b (part of the miR-106b-25 cluster and related to apoptosis), miR-30c and miR-27b was increased upon infection with *Toxoplasma* and *Cryptosporidium*, whereas no change (miR-27b) or even downregulation (miR-106 and miR-30c) was observed after *Plasmodium* challenge. In *Toxoplasma* and *Cryptosporidium*, miR-106b was responsive to NF-κB signaling (Sharma et al., 2009). As similar data was reported for miR-30c, both miRNAs have been considered to be involved in cell immune responses. The members of the miR-27 family, miR-27a and miR-27b, are highly expressed in endothelial cells (Kuehbacher et al., 2007), where they are involved in angiogenesis, and are also present in the central neural system, controlling apoptosis (Jennewein et al., 2010; Chen et al., 2014). Upregulation of the miR-27 family is also observed in the Apicomplexan parasite infection; miR-27a is induced in ECM (El-Assaad et al., 2011) and miR-27b is associated to the TLR4-mediated epithelial anti-microbial defense (Zhou et al., 2012) and apoptosis (Cai et al., 2013).

## Final remarks

One important consideration for the host miRNAs changes discussed above is that in some cases the expression data are from microarray analysis and, thus, it underscores the need for more functional assays to confirm these initial data and the convergence seen on host response. As expected, the immunity response emerges as a common parameter. In the case of malaria, there is evidence for roles for miRNAs in the immune response (Lamonte et al., 2012), however much remains to be explored. The diversity of pathological aspects associated with *Plasmodium* infection makes it difficult to identify specific miRNAs as malaria biomarkers. Currently, a combination of cell sequestration, deformity of RBC and aggregation (Schofield and Grau, 2005; Van der Heyde et al., 2006; Gazzinelli et al., 2014) together with immune responses after *Plasmodium* challenge (Idro et al., 2005; Van der Heyde et al., 2006) are considered as mechanisms underlying severe malaria (Rénia et al., 2012). Besides that, molecular evidences have pointed that severe malaria is related to the expression of a subset of virulence genes that encodes parasite ligands for binding to endothelial cells; through endothelial protein C receptor, for example, in the host brain (Avril et al., 2012; Claessens et al., 2012; Turner et al., 2013). The expression of this subset of virulence genes is controlled by a RNAseII, suggesting virulence factors are also controlled at a molecular level (Zhang et al., 2014). For the time being, only miR-27a fits the bridge between immune signaling pathways and severe malaria, therefore further research should focus on this area.

Moreover, a relevant perspective for miRNAs can be highlighted by the recently decribed potential alternative strategies for cancer therapy which employed mimic and antagonist miRNA molecules in different tumor scenarios (Ma et al., 2010; Wang et al., 2012). In the particular case of protozoan infection, miR-122, already tested for liver tumor suppression (Thakral and Ghoshal, 2015), has a protective function against *Leishmania*. During *Leishmania* infection the loss of hepatic miR-122 is related to an increased parasite burden in patients with visceral leishmaniasis (Ghosh et al., 2013). In this case, therapeutical targeting of miR-122 may be helpful in patients with *Leishmania* infection.

Finally, for all protozoan pathologies, further miRNA research may (i) uncover new biomarkers associated with disease progression, (ii) determine miRNA target genes that can clarify the roles of miRNAs in pathogenesis and (iii) aid in the discovery of new therapeutic targets.

## Author contributions

CJ, CB, and AK contributed to the writing of this review. LA and FC helped with critical revision of the manuscript for intellectual content. All authors read and approved the final manuscript.

## Funding

This work was supported by Fundação de Amparo à Pesquisa do Estado de São Paulo (FAPESP; 2012/16525-2), the Conselho Nacional de Desenvolvimento Científico e Tecnológico (CNPq). CB and AK were sponsored by FAPESP fellowships. FC is a CNPq research fellow.

### Conflict of interest statement

The authors declare that the research was conducted in the absence of any commercial or financial relationships that could be construed as a potential conflict of interest.
