# Supplementary material for: MicroRNAs in the Host-Apicomplexan Parasites Interactions: A Review of Immunopathological Aspects
Source: Front Cell Infect Microbiol. 2016 Feb 2;6:5. doi: 10.3389/fcimb.2016.00005 (PMC4735398; doi:10.3389/fcimb.2016.00005)
Supplement: Supplementary file 1 [file DataSheet1.DOCX]

**
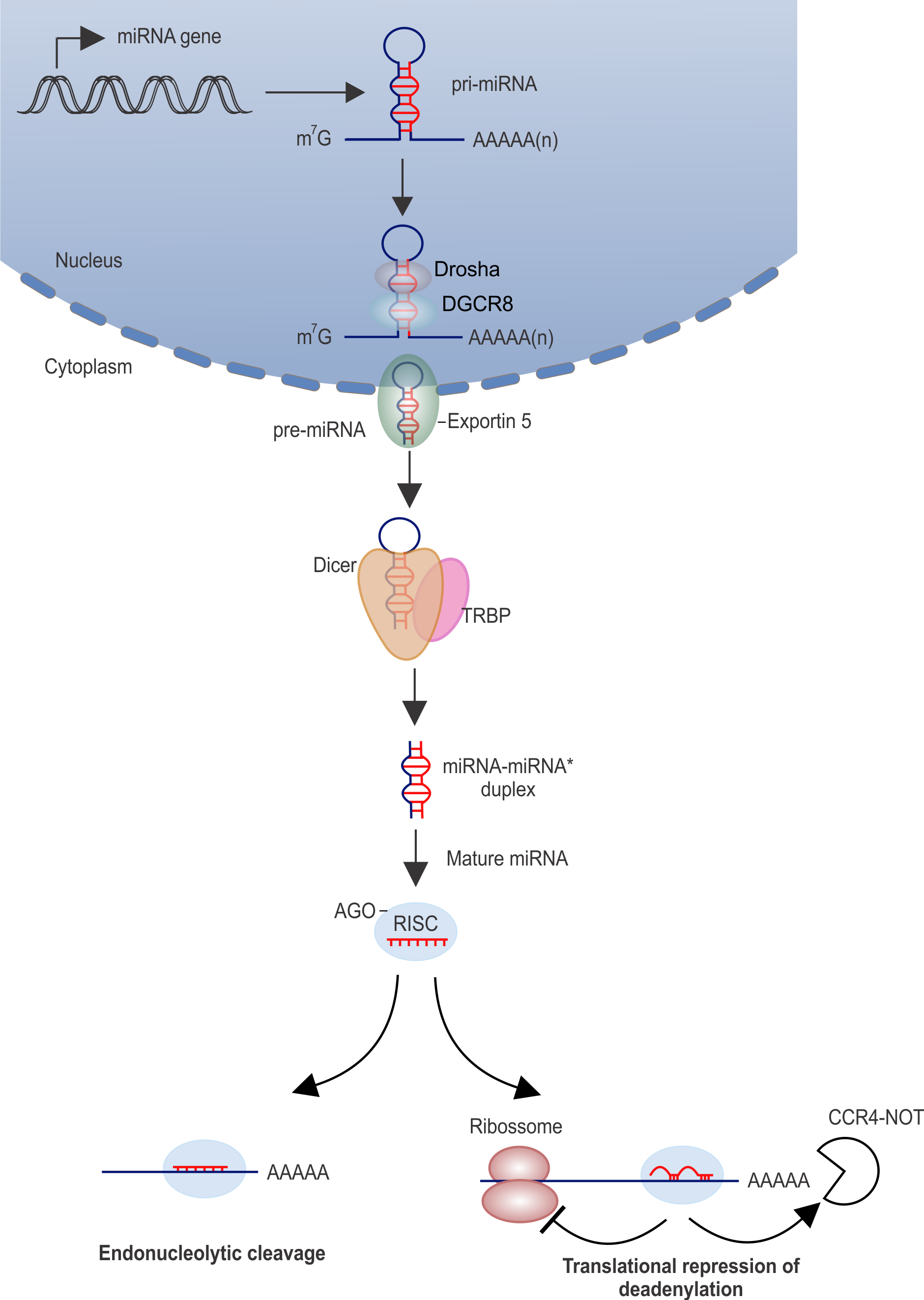
**

**Supplementary Figure 1. MicroRNA biogenesis and mechanisms of action.** In general, microRNAs (miRNAs) are transcribed as long primary transcripts (pri-miRNAs) by RNA polymerase II. The pri-miRNA contains one or more hairpin structures that are specifically spliced by the microprocessor complex comprising Drosha (a nuclear RNase III type endonuclease) and its partner, DGCR8. A pre-miRNA (miRNA precursor) of approximately 70 nucleotides in size is generated, which is subsequently exported into the cytoplasm by exportin 5, a nuclear transport receptor. The pre-miRNA is then recognized by RNAse III Dicer and TRBP (TAR RNA-binding protein) and Dicer cleaves it into smaller duplexes, generating a 20-nucleotide mature miRNA duplex. The functional strand of the mature miRNA is loaded onto a RNA-induced silencing complex (RISC), which contains Argonaute (Ago) proteins. The mature miRNA guides RISC to silence target mRNAs that result from mRNA cleavage, translational repression or deadenylation (removal of polyA tail by deadenylases, such as CCR4-NOT).

m7G: 7-methylguanosine cap.
